# Linking Oxidative Stress and DNA Damage to Changes in the Expression of Extracellular Matrix Components

**DOI:** 10.3389/fgene.2021.673002

**Published:** 2021-07-29

**Authors:** Susana G. Martins, Rita Zilhão, Sólveig Thorsteinsdóttir, Ana Rita Carlos

**Affiliations:** ^1^Centro de Ecologia, Evolução e Alterações Ambientais, Faculdade de Ciências, Universidade de Lisboa, Lisboa, Portugal; ^2^Departamento de Biologia Animal, Faculdade de Ciências, Universidade de Lisboa, Lisboa, Portugal; ^3^Departamento de Biologia Vegetal, Faculdade de Ciências, Universidade de Lisboa, Lisboa, Portugal

**Keywords:** oxidative stress, DNA damage, ECM remodeling, tissue homeostasis, ECM gene expression

## Abstract

Cells are subjected to endogenous [e.g., reactive oxygen species (ROS), replication stress] and exogenous insults (e.g., UV light, ionizing radiation, and certain chemicals), which can affect the synthesis and/or stability of different macromolecules required for cell and tissue function. Oxidative stress, caused by excess ROS, and DNA damage, triggered in response to different sources, are countered and resolved by specific mechanisms, allowing the normal physiological equilibrium of cells and tissues to be restored. One process that is affected by oxidative stress and DNA damage is extracellular matrix (ECM) remodeling, which is a continuous and highly controlled mechanism that allows tissues to readjust in reaction to different challenges. The crosstalk between oxidative stress/DNA damage and ECM remodeling is not unidirectional. Quite on the contrary, mutations in ECM genes have a strong impact on tissue homeostasis and are characterized by increased oxidative stress and potentially also accumulation of DNA damage. In this review, we will discuss how oxidative stress and DNA damage affect the expression and deposition of ECM molecules and conversely how mutations in genes encoding ECM components trigger accumulation of oxidative stress and DNA damage. Both situations hamper the reestablishment of cell and tissue homeostasis, with negative impacts on tissue and organ function, which can be a driver for severe pathological conditions.

## Introduction

Redox reactions are central to ensure correct cell function, allowing a dynamic transfer of electrons across different molecules (McCord, 2000). This occurs, for example, during molecular oxygen metabolism in the mitochondria, which are the central metabolic machinery of cells. Molecular oxygen metabolism is the basis of oxidative phosphorylation, and promotes the production of reactive oxygen species (ROS) (Sabharwal and Schumacker, 2014; Shadel and Horvath, 2015). ROS also play an important role in immune defenses, where innate immune cells produce ROS as part of their pathogen killing strategies (Khan et al., 2018). In fact, neutrophils and some macrophages express myeloperoxidase (MPO), which leads to the generation of different ROS, including hypochlorous acid (HOCl), a potent ROS that is involved in pathogen killing mechanisms (Khan et al., 2018).

However, when in excess, ROS may cause serious damage to different molecules promoting DNA damage, protein oxidation, and lipid peroxidation (McCord, 2000). The deleterious accumulation of ROS may have multiple origins, including the excessive ROS production by the mitochondria, which drives mitochondrial dysfunction, an important trait of a variety of diseases. Mitochondrial dysfunction can be due to a metabolic imbalance (Bhatti et al., 2017), like, for example, in diabetes, where high levels of glucose promote overproduction of ROS (Yaribeygi et al., 2019), or it may also arise as a consequence of mutations in mitochondrial DNA, defective OXPHOS, and alterations in ROS production, which are common phenomena in cancer (Hsu et al., 2016; Porporato et al., 2018). As mentioned above, another example of excess ROS generation is through MPO in neutrophils, which can have a detrimental outcome if it leads to chronic inflammation, a driver of pathology in a variety of diseases (Khan et al., 2018).

DNA damage can be caused by several factors, including by exogenous agents, e.g., UV light, ionizing radiation, as well as by endogenous triggers, such as errors in DNA replication and oxidative stress. DNA replication is a central process during cell division, where accuracy is essential to maintain the genetic information intact (Sancar et al., 2004; Zeman and Cimprich, 2014). However, DNA replication can also be a source of deleterious events leading to DNA damage, in particular, due to aberrant replication fork structures such as breaks associated with replication fork stalling and collapse (Zeman and Cimprich, 2014). Oxidative stress is also an important source of DNA damage (Whitaker et al., 2017), since increased levels of ROS can result in DNA oxidation, particularly in oxidized DNA bases. Depending on the source of DNA damage, a specific machinery is activated to promote successful repair (Sancar et al., 2004; Zeman and Cimprich, 2014). When DNA damage cannot be repaired, cells may undergo programmed cell death or accumulate mutations that drive genomic instability, which can be a trigger for tumorigenesis (Hanahan and Weinberg, 2011).

**Figure 1:**
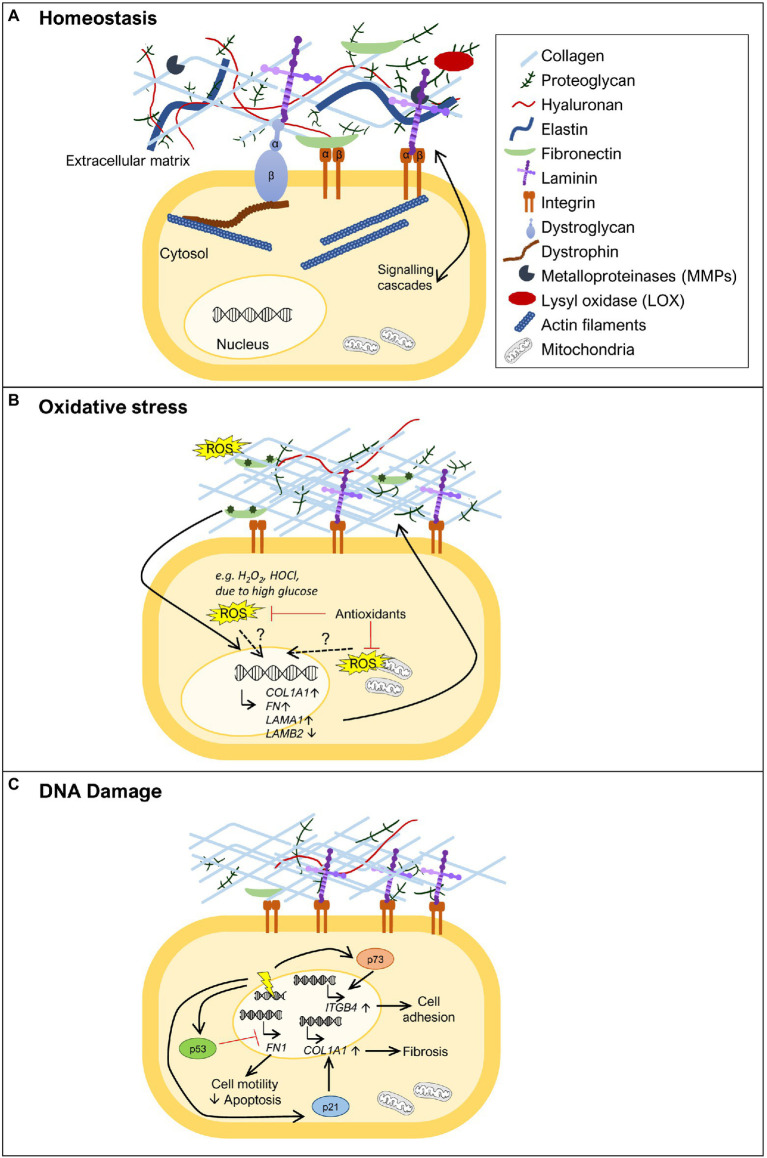
Extracellular matrix (ECM) remodeling upon oxidative stress and DNA damage. **(A)** The ECM is composed of various molecules, such as extracellular glycoproteins (e.g., collagens, fibronectin, and laminins), elastins, proteoglycans, and hyaluronan. These components are in close communication with the cell *via* transmembrane proteins (e.g., integrins, syndecans, and dystroglycan) that anchor different ECM components to the cell, activating various signal transduction cascades. Specific tissues have specialized ECMs and cell surface receptors, as can be observed for skeletal muscle, where the dystrophin-glycoprotein complex anchors laminins and links them to the actin cytoskeleton *via* dystrophin. ECM remodeling is carried out through the action of metalloproteinases (MMPs) and lysyl oxidase (LOX). **(B)** ECM remodeling can be promoted by oxidative stress. In the presence of increased reactive oxygen species (ROS) levels [e.g., diabetes, high H_2_O_2_, hypochlorous acid (HOCl), or HOCl-modified fibronectin], some ECM genes become more highly expressed, such as fibronectin (*FN1*), collagen α1 chain (*COL1A1*), and laminin α1 chain (*LAMA1*), while others show decreased expression, as observed for laminin β2 chain (*LAMB2*). The exact mechanism(s) by which oxidative stress induces expression of ECM genes remains to be fully uncovered. The role of oxidative stress is also revealed by treatment with antioxidants where reduction in the levels of ROS normalizes ECM composition. **(C)** DNA damage (represented as a yellow electric ray symbol on top of double stranded DNA) is another insult that triggers changes in the ECM. The DNA damage responsive protein p53 has been implicated in the downregulation of *FN1* expression, promoting cell motility and suppressing apoptosis. Another important player in this context is p21. This negative regulator of the cell cycle has been shown to induce *COL1A1* expression and consequently lead to collagen deposition and fibrosis. The transcription factor p73, a p53 family member, is also a key protein in ECM remodeling in the response to DNA damage. Upon activation, p73 has been demonstrated to directly promote the transcription of *ITGB4*, encoding integrin β4, and consequently promoting cell adhesion. For simplicity, not all ECM components are shown in the figures, just the ones that are relevant to illustrate the phenomenon being highlighted.

Even though, dedicated machineries exist to cope with both oxidative stress and DNA damage, when these insults occur, they often lead to a variety of cellular and extracellular changes, which are important to ensure that cells return to their normal physiological equilibrium. One of these changes is extracellular matrix (ECM) remodeling. The ECM provides support to tissues and contributes to maintain their appropriate physiological environment (Frantz et al., 2010; Bonnans et al., 2014). It consists of a network of extracellular glycoproteins (e.g., collagens, fibronectin, and laminins), elastins, proteoglycans, hyaluronan, and a number of other less well characterized ECM components, collectively termed matrisome (Hynes and Naba, 2012; Naba et al., 2016; Figure 1A). ECM components can be directly linked to the cell surface and the underlying cytoskeleton, allowing a rapid and precise communication between the cells and the ECM. The communication between the extracellular and intracellular environments is mediated by transmembrane proteins, such as integrins, syndecans, and dystroglycan (Moore and Winder, 2012; Afratis et al., 2017; Bachmann et al., 2019). These transmembrane proteins bind directly to specific domains of ECM molecules on the extracellular side, and to cytoskeletal proteins and different kinases on the intracellular side, thus allowing the transmission of signaling cascades. In addition, the ECM can also act as a route or reservoir for different molecules with important autocrine and paracrine functions that include cytokines, chemokines, and growth factors, which modulate a variety of different cellular processes, such as cell adhesion, migration, differentiation, proliferation, survival, and apoptosis (Bowers et al., 2010; Bonnans et al., 2014).

Extracellular matrix remodeling is a normal process that occurs during embryonic development, allowing the progressive formation of tissues and organs, and continues throughout adulthood in order to ensure the balance of organismal homeostasis (Rozario and DeSimone, 2010; Bonnans et al., 2014). It is also an important player in tissue healing and repair but, when not properly regulated, it can result in the loss of normal tissue structure and the development of different pathological conditions. Examples of such conditions are (i) fibrosis, where extensive ECM deposition and scar formation hamper normal tissue and organ function (Herrera et al., 2018; Li et al., 2018), (ii) diabetes, where ECM remodeling is negatively affected in several aspects of the disease, including in the impaired healing of chronic diabetic wounds and in diabetes-associated fibrosis (Falanga, 2005; Law et al., 2012; Ahmad et al., 2020), and (iii) cancer, where several components involved in ECM remodeling have their specific functions hijacked by cancer cells, enabling their proliferation, epithelial-to-mesenchymal transition, and migration (Bonnans et al., 2014; Winkler et al., 2020). Interestingly, diseases characterized by extensive ECM remodeling often involve oxidative stress and DNA damage, either as the triggers or as a consequence of their pathophysiology. This is the case of the aforementioned pathologies: fibrosis, a pathological condition marked by high levels of oxidative stress and a direct implication of DNA damage (Cheresh et al., 2013; Li et al., 2018); diabetes, which is associated with high levels of blood glucose that in turn lead to increased ROS levels (Yaribeygi et al., 2019); and cancer in which both DNA damage and oxidative stress play an important role in disease progression (Luo et al., 2009; Reuter et al., 2010; Hanahan and Weinberg, 2011). On the other hand, several diseases due to mutations in ECM components or proteins linking the ECM to the cell cytoskeleton have been shown to display increased oxidative stress and DNA damage in the affected tissues (Rando, 2002; Irwin et al., 2003; Rodriguez and Tarnopolsky, 2003; Millay et al., 2008; Shkryl et al., 2009; Menazza et al., 2010; de Oliveira et al., 2014; Sorato et al., 2014; Fontes-Oliveira et al., 2017; Gremminger et al., 2019; Kölbel et al., 2019; Moore et al., 2020). This is particularly striking when considering the muscular dystrophies, but other examples also exist.

It is essential to point out that ECM remodeling does not only involve the synthesis and deposition of ECM molecules, which transmit signals to cells through cell surface receptors. The action of specific proteins that degrade ECM components, including metalloproteinases (MMPs), and enzymes that shape ECM structure, such as lysyl oxidase (LOX), also play a crucial role (Bonnans et al., 2014; Shimoda and Khokha, 2017; Laczko and Csiszar, 2020; Figure 1A). Indeed, the importance of MMPs and their inhibitors, tissue inhibitors of metalloproteinases (TIMPs), in the response to oxidative stress and DNA damage has been extensively addressed elsewhere (Mehta et al., 2006; Kandasamy et al., 2010; Reuter et al., 2010; Sanchez-Valle et al., 2012; Broustas and Lieberman, 2014; Kumari et al., 2018) and are beyond the scope of this review.

Considering the body of work showing the deleterious effects of oxidative stress and DNA damage in the context of a variety of diseases on the one hand, and on the other hand, the impact of changes in the ECM for disease onset and progression, this review aims to examine the potential links between oxidative stress/DNA damage and the ECM in the context of disease development. We explore studies showing how oxidative stress and DNA damage affect the synthesis of ECM molecules, focusing on the major, and best studied, glycoproteins of the ECM, namely collagens, fibronectin, and laminins (Hynes and Naba, 2012; Naba et al., 2016). We also examine the inverse situation, where mutations in genes encoding some of these key ECM glycoproteins or proteins that link the ECM to the cell cytoskeleton trigger an accumulation of oxidative stress and DNA damage. This dual approach aims to address to what extent oxidative stress and DNA damage are part of the molecular processes that control, and are controlled by, the ECM, and through this perspective, we may deepen our understanding of how they contribute to disease development and progression.

## The Impact of Oxidative Stress on ECM Remodeling

Analysis of NCBI Gene Expression Omnibus database for stress responses in HeLa cells showed that several different forms of stress, including oxidative stress, lead to changes in the expression of genes encoding structural and signaling components of the ECM (Chovatiya and Medzhitov, 2014). Here, we review the literature reporting when oxidative stress affects the synthesis or structure of the best studied ECM glycoproteins, namely collagens, fibronectin, and laminins.

Several studies have shown that the synthesis and/or the stability of several collagens, including collagen I, the most abundant fibrous protein of the ECM, are regulated by oxidative stress (Figure 1B). However, the exact role played by oxidative stress in the regulation of collagen synthesis is still not fully understood, and apparent contradictory results have been described. Siwik et al. (2001) showed that oxidative stress can inhibit collagen I and IV mRNA expression, while other studies have shown that oxidative stress can also lead to increased collagen expression (Iglesias-De La Cruz et al., 2001; Zhao et al., 2008; Guo et al., 2018; Kazakov et al., 2018). This apparent discrepancy might be explained by the amount of oxidative stress in the tissue, as suggested by Liu et al. (2016). In this work, human uterosacral ligament-derived fibroblasts were treated with a low concentration of H_2_O_2_, an important ROS, which led to a decrease in *COL1A1* (gene codifying for collagen type I α1 chain) expression. In contrast, when these cells were exposed to a higher concentration of H_2_O_2_, their *COL1A1* expression increased (Liu et al., 2016). This suggests that mild oxidative stress, associated with normal metabolism or small insults, may promote the reduction of collagen levels, whereas high levels of oxidative stress may favor collagen accumulation. Collagen deposition is required for wound healing, but if in excess, can also account for a tissue injury status like fibrosis. In support of this observation, several studies have shown that the presence of elevated levels of oxidative stress lead to increased collagen I and III levels contributing to myocardial fibrosis (Zhao et al., 2008; Sriramula and Francis, 2015; Kazakov et al., 2018). In addition, treatment with antioxidants in the context of cardiac fibrosis has been shown to reduce collagen levels (Zhao et al., 2008; Guo et al., 2018), further suggesting a direct effect between increased oxidative stress and collagen deposition. Glucose deregulation is another mechanism known to cause oxidative stress, as observed for diabetes (Yaribeygi et al., 2019). Kidney glomerular mesangial cells cultured with high levels of glucose show increased collagen IV synthesis (Ayo et al., 1991). Moreover, the synthesis of collagen I and III is increased in hearts of experimental diabetic rats, a condition that was countered by antioxidant treatment (Guo et al., 2018). The regulation of collagen synthesis by ROS may not only occur at the transcriptional level. It is well established that the antioxidant vitamin C is a cofactor of prolyl hydroxylase and lysyl hydroxylase, required for post translational modifications of procollagen and consequently in the correct formation of the mature collagen triple helix (Frantz et al., 2010; Bonnans et al., 2014). To corroborate the relationship between collagen levels and oxidative stress, it has recently been suggested that vitamin C, due to its role as an antioxidant, could improve healing associated with musculoskeletal diseases, by reducing the levels of ROS associated with the inflammatory response (DePhillipo et al., 2018). Even though, pre-clinical studies have shown that vitamin C can reduce ROS and promote tissue healing *via* collagen I synthesis during bone fracture recovery and in ruptured tendons, more studies in a clinical setting are needed to assess its therapeutic benefit (DePhillipo et al., 2018).

The expression or stability of fibronectin has also been linked to oxidative stress. Fibronectin (*FN1*) expression has been shown to be positively regulated in the presence of different sources of oxidative stress (Iglesias-De La Cruz et al., 2001; Siwik et al., 2001; Lee et al., 2004; Nybo et al., 2018; Figure 1B). Oxidative stress, due to high glucose levels, increases the expression of *FN1 in vitro* (Ayo et al., 1991; Lee et al., 2004; Lin et al., 2006), as well as *in vivo*, in the context of diabetes (Lin et al., 2006). ROS reduction by treatment with the ROS scavenging enzyme superoxide dismutase (SOD) led to a reduction in fibronectin levels in diabetic rats and to less kidney damage (Lin et al., 2006). Furthermore, HOCl generated through MPO activity in neutrophils has been shown to induce fibronectin modifications, such as tyrosine chlorination and dichlorination and oxidation of different residues, which reduced cell adhesion and increased proliferation of human coronary artery smooth muscle cells *in vitro* (Nybo et al., 2018). The HOCl-induced modifications of fibronectin also led to changes in the expression of ECM genes by these smooth muscle cells, including a significant upregulation of *FN1* and *LAMA1* and downregulation of *LAMB2* expression (Nybo et al., 2018; Figure 1B). *LAMA1* codifies the laminin α1 chain, which is normally not present in the basement membrane of smooth muscle cells, while downregulation of *LAMB2* codifying the laminin β2 chain indicates that cells are prevented from maintaining β2 laminins, characteristic of mature smooth muscle basement membranes (Yousif et al., 2013). These data indicate that cells attempt to remodel their ECM in response to their oxidant-altered fibronectin substrate and associated effects on cell adhesion and proliferation.

Altogether, these various studies show that oxidative stress causes changes in both ECM gene expression and in ECM structure which correlate with tissue damage.

## The Impact of DNA Damage on ECM Remodeling

It is well established that DNA integrity is essential for gene expression and for the correct transmission of genetic information to daughter cells during mitosis. It is not surprising that DNA damage is one of the most deleterious forms of injury inflicted on cells (Sancar et al., 2004; Branzei and Foiani, 2008). Here, we will consider how DNA damage, independently of the source (e.g., ROS, radiation, and mutagens), can directly or indirectly affect the expression of genes encoding either ECM glycoproteins or proteins linking the ECM to the cell cytoskeleton.

A central protein involved in the DNA damage response is the tumor suppressor p53, which, when activated, either induces cell cycle arrest to allow for DNA repair or, if the damage inflicted is too large, promotes, for example, apoptosis (Sancar et al., 2004; Branzei and Foiani, 2008; Williams and Schumacher, 2016). p53 has been implicated in the regulation of *FN1* expression. Depletion of p53 was shown to increase *FN1* mRNA expression, leading to increased cell motility and decreased apoptosis (Yokoi et al., 2020; Figure 1C), while treatment with the p53 activator RITA caused a decrease in fibronectin levels (You et al., 2017). These studies thus indicate p53 as a negative regulator of *FN1* expression. In accordance with this notion, overexpression of wild type p53 in ovarian carcinoma cells was able to repress the activity of the *FN1* promoter, while a mutant p53 failed to do so (Yokoi et al., 2020), showing that p53 can act as a transcription factor directly regulating *FN1* transcription. Another indication of an effect of DNA damage on the expression of ECM components came from a study on the CDK inhibitor p21 (Yosef et al., 2017), a negative regulator of the cell cycle, which can be induced by p53 and drive cell cycle arrest (Williams and Schumacher, 2016). This study revealed that in p21 knockout mice, senescent cells were eliminated, and liver fibrosis was alleviated mainly *via* transcriptional downregulation of collagen type I α1 (*COL1A1*; Yosef et al., 2017; Figure 1C). In fact, p21 knockout reduced *Col1a1* expression, prevented collagen deposition and consequently fibrosis, in mice treated with carbon tetrachloride, a fibrogenic agent known to cause DNA damage (Yosef et al., 2017). These observations suggest that one of the actions of p21 is to increase *COL1A1* expression in response to DNA damage. However, further studies are needed to assess whether these described effects of p53 and p21 on *FN1* and *COL1A1* expression, respectively, are cell type specific and exactly how the pathways involved control cell survival, senescence, and apoptosis.

Integrins provide yet another link between ECM structure and DNA damage-induced apoptosis (Hoyt et al., 1996; Sethi et al., 1999; Hodkinson et al., 2006; Jinushi et al., 2012; Ahmed et al., 2018; Kowalski-Chauvel et al., 2018; Hou et al., 2019; Jung et al., 2019; Naci et al., 2019; Li et al., 2021). Nevertheless, the connection between adhesion and apoptosis is not linear and integrin binding to the ECM can either promote or prevent cells, in particular cancer cells, from undergoing apoptosis. It is possible that regulation mediated by factors involved in the DNA damage response, such as p53 and p21, control the expression of integrins or ECM components and therefore modulate adhesion. Indeed, it has been shown that p73, a p53 family member that can also be activated in response to DNA damage, directly promotes transcription of *ITGB4*, encoding integrin β4, therefore acting as a positive regulator of cell adhesion (Xie et al., 2018; Figure 1C).

Collectively, this suggests that central DNA damage response factors might be able to directly control the expression of ECM components and integrins, hence being able to regulate cell survival and apoptosis.

## Mutations in Genes Encoding ECM Components Trigger Oxidative Stress and DNA Damage

As discussed above, the notion that oxidative stress and DNA damage result in alterations in the ECM in the context of different diseases has been gaining ground. However, does the inverse occur? Does a dysfunctional ECM induce oxidative stress and DNA damage in the cells it harbors? Indeed, some observations suggest that this is the case. For example, culture of human dermal fibroblasts in fragmented collagen matrices, resulted in elevated levels of ROS in these cells compared to fibroblasts cultured in intact collagen matrices (Fisher et al., 2009). Here, we explore the literature on diseases caused by mutations in genes encoding ECM components and proteins that link the ECM to the cytoskeleton, more specifically collagens, laminin 211, and dystrophin, to assess whether they are linked to oxidative stress and/or DNA damage.

### Collagen Deficiency

**Figure 2:**
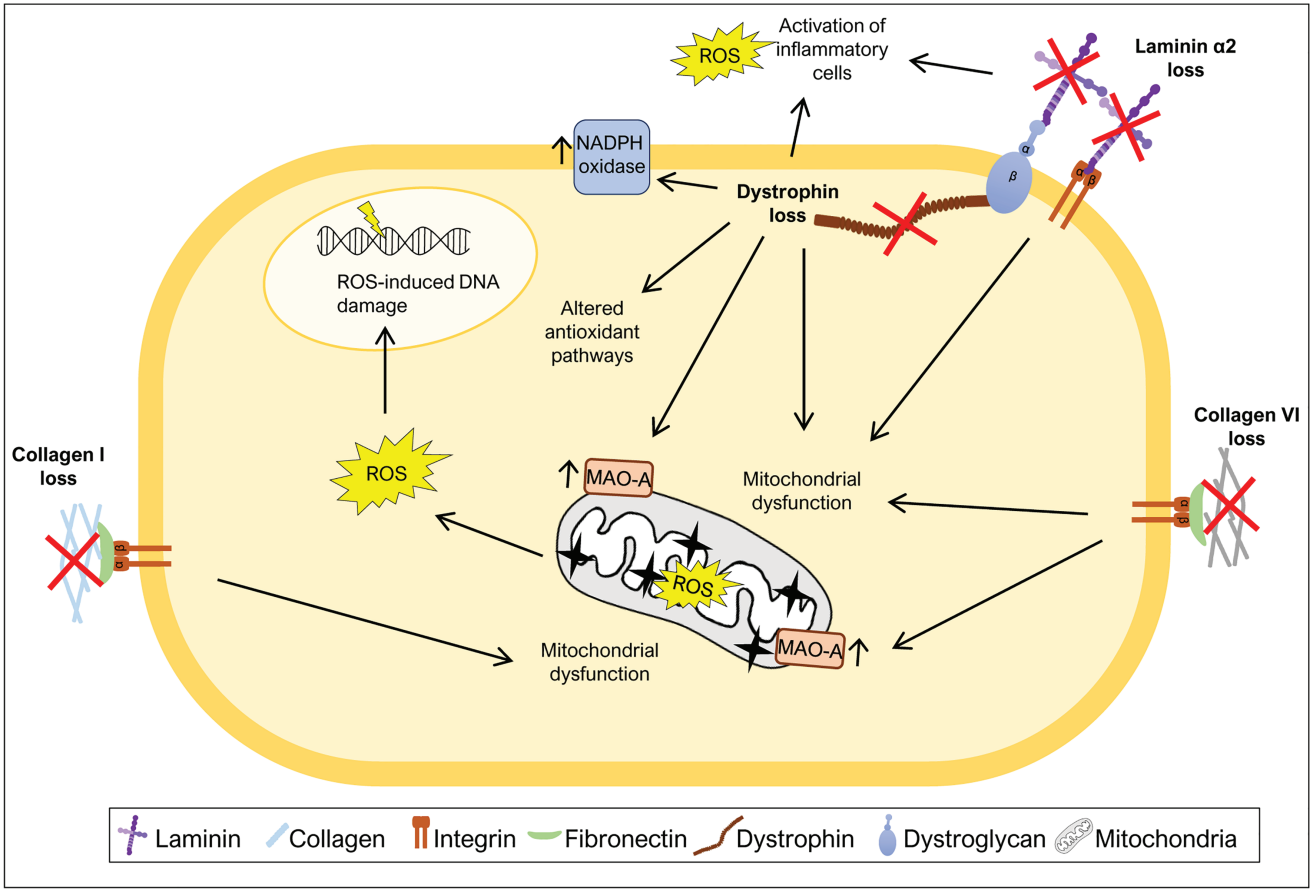
Mutations in ECM components increase oxidative stress. Many diseases caused by mutations in ECM components or components that link ECM to the cytoskeleton have been associated with generation of excess ROS levels. The loss of collagen I, collagen VI, laminin α2, or dystrophin was shown to induce mitochondrial dysfunction, a major source of ROS. Additionally, high ROS levels due to either increased activity of monoamine oxidase A (MAO-A) or NADPH oxidase have also been associated with the loss of specific ECM or ECM-related components. Loss of laminin α2 or dystrophin leads to increased levels of ROS as a consequence of the activation of inflammatory cells. Dystrophin loss was also shown to promote changes in the activity of antioxidant pathways and ROS-induced DNA damage.

Collagen I is present in a variety of connective tissues (Frantz et al., 2010; Bonnans et al., 2014). Mutations in *COL1A1* and *COL1A2* genes, encoding the α1 and α2 chains of collagen I, respectively, cause osteogenesis imperfecta, a disease characterized by brittle bones, short stature, and muscle weakness (van Dijk et al., 2011; Marini et al., 2017). Recently, a study using the mouse model of osteogenesis imperfecta, the *Col1a2^oim^* mouse, also known as *oim*/*oim* mouse, revealed that the muscles of mice homozygous for the mutation display a mitochondrial dysfunction in that they have decreased levels of electron transport chain complex IV, mitochondrial encoded cytochrome oxidase I, energy production, citrate synthase activity and respiration, and elevated levels of PGC1α (Gremminger et al., 2019), all markers of mitochondrial dysfunction (Adhihetty et al., 2009; Li et al., 2020; Melouane et al., 2020; Figure 2). These results suggest that mitochondrial dysfunction is an important feature of the muscles of *oim*/*oim* mice, but the exact mechanism that links collagen I deficiency to mitochondrial dysfunction remains to be determined. Moreover, excessive ROS production in the context of collagen I deficiency may also contribute to the exacerbation of osteogenesis imperfecta pathology in bone. Indeed, high ROS generation is associated with inhibition of new bone formation, suggesting that antioxidant treatment may be useful to treat diseases with bone loss (Wauquier et al., 2009).

Collagen VI is a type of collagen mainly found in muscles, tendons, and skin (Bönnemann, 2011). Mutations in the *COL6A1*, *COL6A2*, and *COL6A3* genes, encoding chains of collagen VI, are associated with Ullrich congenital muscular dystrophy and Bethlem myopathy, a severe and mild form of collagen VI deficiency, respectively (Bönnemann, 2011; Bernardi and Bonaldo, 2013). Collagen VI deficiency has also been correlated with oxidative stress. Studies using a mouse model of collagen VI deficiency, *Col6a1*^−/−^ mice, revealed elevated levels of ROS, mitochondrial dysfunction and elevated levels of monoamine oxidase A (MAO-A) (Irwin et al., 2003; Menazza et al., 2010; Sorato et al., 2014; Figure 2), one of the major contributors to ROS production in the mitochondria (Canton et al., 2014). After treatment with MAO inhibitor, muscles from *Col6a1*^−/−^ mice showed a decrease in ROS levels when compared to untreated mice (Menazza et al., 2010; Sorato et al., 2014), highlighting the enhanced production of ROS by these enzymes when collagen VI is absent. Consistent with this, treatment with a MAO inhibitor (Menazza et al., 2010; Sorato et al., 2014) and treatment with a mitochondrial permeability transition pore inhibitor (Irwin et al., 2003; Merlini et al., 2008; Palma et al., 2009), reduced the apoptotic phenotype and improved mitochondrial function in *Col6a1*^−/−^ mice and patients with collagen VI deficiency. Altogether, these studies reinforce the idea that the absence of collagen VI drives excess ROS production in the mitochondria, potentially contributing to the observed muscle fiber damage.

### LAMA2 Deficiency

The *LAMA2* gene encodes the laminin α2 chain of laminin 211. Mutations in *LAMA2* lead to a congenital muscular dystrophy (LAMA2-CMD; also known as merosin-deficient congenital muscular dystrophy type 1A, MDC1A) which is characterized by muscle weakness, fibrosis, and chronic inflammation (Gawlik and Durbeej, 2011; Yurchenco et al., 2018). Recently, different mouse models of LAMA2-CMD (the *dy^2J^/dy^2J^* and *dy^3K^/dy^3K^* mouse models) and biopsies from LAMA2-CMD patients were used to address the impact of oxidative stress on this condition (de Oliveira et al., 2014; Fontes-Oliveira et al., 2017; Gawlik et al., 2019; Kölbel et al., 2019; Harandi et al., 2020). These studies indicate that the muscles of *Lama2*-deficient mice and LAMA2-CMD patients display an increase in oxidative stress both in early and more advanced stages of the disease, pointing to an important role of oxidative stress throughout disease progression (Gawlik et al., 2019; Harandi et al., 2020). In fact, *dy^3K^/dy^3K^* mice show high levels of oxidative stress in quadriceps at least from post-natal day 7 and increased *Gclm* and *Sqstm1* expression, markers of oxidative stress, at post-natal day 21 (Gawlik et al., 2019). Analysis of muscles from 3-week-old *dy^2J^/dy^2J^* mice and muscle biopsies from LAMA2-CMD patients (age ranging between 22 days and 29 years) also confirmed the presence of elevated levels of ROS in both cases (Harandi et al., 2020). Moreover, the treatment of *dy^2J^/dy^2J^* mouse quadriceps and triceps muscles with the antioxidant *N*-acetylcysteine (NAC) partially rescued the motor function of *dy^2J^/dy^2J^* mice, reduced the number of apoptotic fibers, lowered the levels of inflammation markers and prevented fibrosis (Harandi et al., 2020). Vitamin E, another antioxidant, applied to the same mouse model reduced the number of apoptotic fibers and attenuated inflammation in quadriceps muscles, although, it did not improve motor function or reduce fibrosis (Harandi et al., 2020). Nevertheless, both antioxidants were able to improve *dy^2J^/dy^2J^* skeletal muscle morphology, in particular, by normalizing the proportion of small fibers, decreasing the number of fibers with centrally located nuclei, and decreasing ROS production (Harandi et al., 2020).

Mitochondrial dysfunction, closely associated with elevated oxidative stress, also plays a central role in LAMA2-CMD pathology. In fact, it was observed that *Lama2*-deficient mice present signs of mitochondrial swollenness (Millay et al., 2008) and downregulation of a variety of mitochondria specific genes encoding components of the electron transport chain/oxidative phosphorylation (de Oliveira et al., 2014; Fontes-Oliveira et al., 2017; Figure 2). Likewise, muscles or muscle-derived cells from LAMA2-CMD patients were described as having an abnormal membrane potential (Fontes-Oliveira et al., 2017), impaired mitochondrial function and bioenergetic status and downregulation of a variety of mitochondrial related genes (Fontes-Oliveira et al., 2017; Kölbel et al., 2019). Altogether, these results support the hypothesis that oxidative stress and mitochondrial dysfunction are hallmarks of LAMA2-CMD.

### Dystrophin Mutations

Dystrophin is part of the dystrophin-glycoprotein complex which links cytoplasmic dystrophin to laminin 211 in the muscle fiber basement membrane, a link that is responsible for conferring cellular stability during skeletal muscle contractions (Ervasti and Campbell, 1993). Mutations in the *DMD* gene, encoding dystrophin, can cause Duchenne muscular dystrophy (DMD), characterized by muscle weakness, inflammation, and fibrosis (Grounds et al., 2020). Several mechanisms are known to contribute to DMD pathology, including oxidative stress (Rando, 2002; Grounds et al., 2020). Early studies of the disease showed that DMD muscles display oxidative stress features, such as increased lipid peroxidation (Hunter and Mohamed, 1986) and increased levels of oxidative stress responsive enzymes (Austin et al., 1992). Moreover, *in vivo* and *in vitro* analyzes of *mdx* mice, the most widely used DMD model, showed an increased susceptibility to muscle injury caused by free radicals (Disatnik et al., 1998; Rando et al., 1998), suggesting a deficiency in oxidative stress responses in *mdx* mice. During the last decades, the oxidative stress hypothesis has been gathering support with several studies showing high levels of ROS in muscles of *mdx* mice (Ragusa et al., 1997; Williams and Allen, 2007; Segatto et al., 2020) and DMD patients (Haycock et al., 1997; Rodriguez and Tarnopolsky, 2003). The excessive production of ROS in DMD patients and *mdx* mice may have multiple sources, including activation of inflammatory cells (Whitehead et al., 2008; Grounds et al., 2020), increased activity of NADPH oxidase (Williams and Allen, 2007; Whitehead et al., 2010), mitochondrial dysfunction (Shkryl et al., 2009; Menazza et al., 2010), and alterations in antioxidant pathways, including, for example, the elevated levels of oxidative stress sensor nuclear factor erythroid 2-related factor 2 (Nrf2) (Petrillo et al., 2017) and overactivation of the nuclear factor κB (NF-κB) pathway (Kumar and Boriek, 2003; Monici et al., 2003; Figure 2). Using antioxidizing drugs, such as vitamin C or SOD, and treatment with the antioxidant NAC reduced the levels of ROS in cardiac (Williams and Allen, 2007) and skeletal muscles (Whitehead et al., 2008) of *mdx* mice. It also reduced the expression of markers of inflammation and fibrosis (Williams and Allen, 2007), as well as, the number of apoptotic fibers (Whitehead et al., 2008). This observed rescue of the DMD phenotype using antioxidant treatments suggests a possible role of ROS in the inflammation, fibrosis, and apoptosis characteristic of DMD.

Elevated levels of NADPH oxidase and its regulator caveolin-3 is also a hallmark of *mdx* muscles (Williams and Allen, 2007; Whitehead et al., 2010; Petrillo et al., 2017; Segatto et al., 2020). NADPH oxidase produced in skeletal muscles fibers has been shown to be a major source of stretch-induced ROS in *mdx* mice (Shkryl et al., 2009; Whitehead et al., 2010), since it allows the generation of the free radical superoxide. Furthermore, NAC treatment reduced caveolin-3 expression and NF-κB activation in skeletal muscles from *mdx* mice (Whitehead et al., 2008). Another important player in DMD pathology is mitochondrial dysfunction (Menazza et al., 2010; Moore et al., 2020). Elevated levels of MAO were reported in *mdx* mice (Menazza et al., 2010), as well as, reduced mitochondrial DNA copy number (Moore et al., 2020). Inhibition of MAO reduced tropomyosin oxidation (an oxidation marker), normalized fiber size, and reduced tissue inflammation and apoptosis (Menazza et al., 2010).

Reactive oxygen species pose various risks for the integrity of different macromolecules including DNA. In keeping with this notion, DMD patients present high levels of DNA damage associated with increased oxidative stress (Rodriguez and Tarnopolsky, 2003) and cells derived from DMD patients are more sensitive to DNA-damaging agents than control cells (Robbins et al., 1984). More recently, Jelinkova et al. (2019) generated dystrophin-deficient human pluripotent stem cell (DMD hPSC) and showed that these cells have high levels of ROS, which leads to increased levels of ROS-induced DNA damage.

Although, the precise mechanisms and direct or indirect actions and targets are not yet clarified, all these studies sustain the notion that mutations in the genes encoding ECM components, or associated proteins, may trigger oxidative stress and DNA damage. Importantly, some studies, especially those focusing on muscular dystrophies, have raised the question whether oxidative stress constitutes a primary or a secondary event in disease progression (Rando, 2002; Moore et al., 2020). In most cases, studies are conducted during the active phase of the disease, making it impossible to distinguish between these two events. Thus, in the context of mutations in genes encoding ECM components or proteins that link the ECM to cytoskeleton, it is unclear whether oxidative stress acts as a cause, a consequence or both, of disease progression. Hence, more studies are needed to fully understand how oxidative stress contributes to each phase of the disease. Taking into account the link between oxidative stress and DNA damage, it is likely that oxidative stress induced DNA damage may not be limited to DMD (Robbins et al., 1984; Rodriguez and Tarnopolsky, 2003; Jelinkova et al., 2019), but may also be a feature of the other diseases discussed here. The concomitant presence of oxidative stress and DNA damage can act as an important driving force for disease progression. Therefore, understanding the exact mechanisms behind the accumulation of oxidative stress in each phase of these diseases and determining the cellular consequences of increased ROS in every phase is crucial to design targeted therapies for these devastating conditions.

## Discussion

The ECM is an intricate network of different molecules that communicate directly to the intracellular space through cell surface receptors and downstream signaling cascades. It is becoming increasingly clear that altered expression of one of these molecules may perturb this fine-tuned communication, triggering a domino effect, which severely compromises tissue integrity. In this review, we examined several examples, where increased oxidative stress and DNA damage alter the expression of genes encoding key ECM components, and conversely, where mutations in genes encoding some of the best studied ECM glycoproteins and proteins linking the ECM to the cell cytoskeleton elicit an increase in oxidative stress and DNA damage. The crosstalk between these two processes, and their major impact on tissue integrity and function, highlights the necessity to study the molecular nature of this relationship further, opening the possibility of identifying new candidate pathways as targets for therapy.

In response to oxidative stress and DNA damage, regulation of ECM remodeling may occur at different levels, including (i) transcriptional, *via* modulation of different transcription factors, (ii) posttranslational, by the action of MMPs, TIMPs, and LOX, which control the degradation of ECM components, and (iii) at the network level, where changes in an ECM components or molecules that bridge the ECM to the cell cytoskeleton, may trigger a chain reaction that can compromise tissue integrity. Here, we discuss how oxidative stress and DNA damage could influence or be influenced by the expression of genes encoding ECM components or proteins linking to the cell cytoskeleton and dissect out which pathways may be required for this regulation.

**Figure 3:**
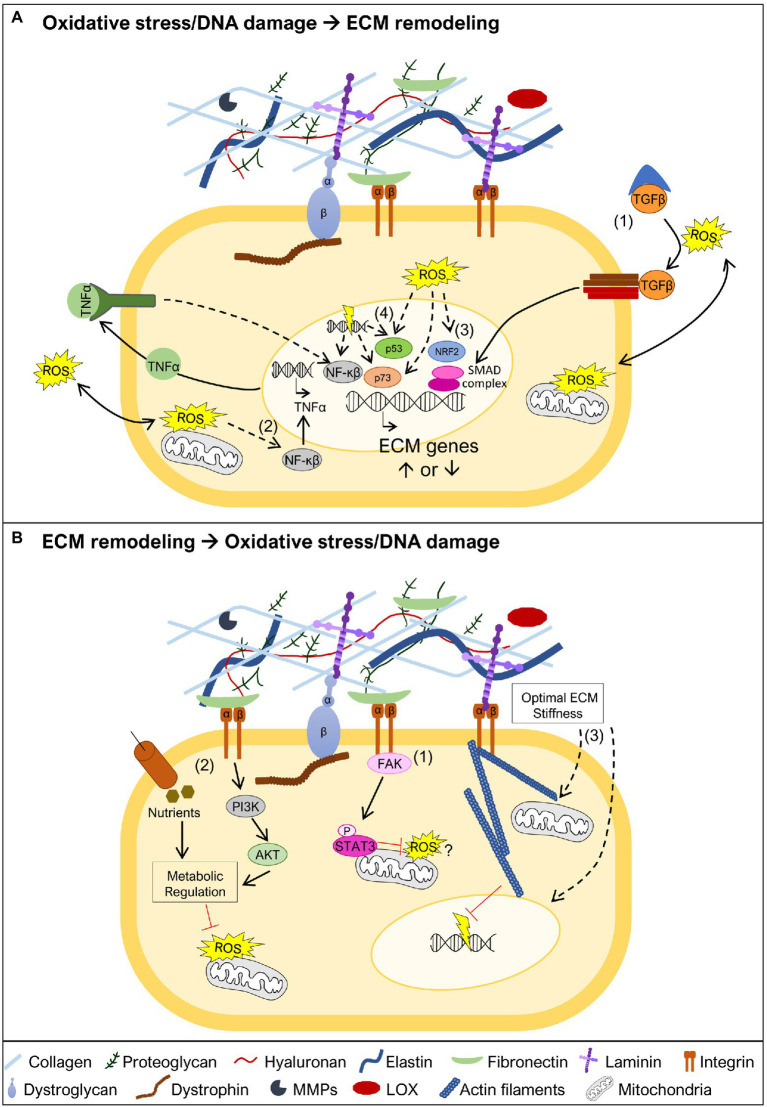
Oxidative stress and DNA damage links to ECM remodeling. **(A)** Oxidative stress and DNA damage may trigger ECM remodeling by different mechanisms. One possible mechanism is associated with ROS-mediated release of TGFβ from the large latent complex, allowing it to bind to the TGFβ receptor (1). This promotes the activation of the SMAD transcriptional complex, known to promote transcription of ECM genes. Another possible mechanism involves the activation of the NF-κβ transcription factor (2). This may occur in the presence of oxidative stress leading to NF-κβ mediated transcription of TNFα, which in turn further promotes NF-κβ activation and transcriptional regulation of ECM genes. NF-κβ can also be activated by DNA damage and control the transcription of ECM target genes. The oxidative stress sensor nuclear factor erythroid 2-related factor 2 (NRF2; 3), may also have a crucial role in the transcriptional regulation of ECM genes. The transcription factors p53 and p73, which regulate ECM gene expression, are both known targets of oxidative stress and DNA damage (4). **(B)** ECM stability is important to prevent the generation of oxidative stress and DNA damage. The integrin-focal adhesion kinase (FAK) pathway promotes mitochondrial function, *via* phosphorylation of STAT3, which is implicated in the reduction of ROS and maintenance of mitochondrial membrane potential (1). Maintaining ECM integrity and cell adhesion contributes to sustain nutrient uptake and phosphatidyl-inositol-3-kinase (PI3K)/AKT signaling, together reinforcing metabolic regulation, and preventing accumulation of ROS (2). Biomechanical properties of the ECM are also critical to maintain cell structure (3). An appropriate ECM stiffness maintains mitochondrial dynamics, possibly preventing ROS production, and nuclear structure, supporting genomic integrity and avoiding DNA damage. Dashed lines represent pathways that may involve one or more intermediate players.

TGF-β signaling is one of the key regulators of ECM production (Hinz, 2015; Tu and Quan, 2016), *via* the activation of Smad transcription factors, which regulate the transcription of ECM genes (Figure 3A), for example, collagens and fibronectin, but also ECM proteolytic enzymes, such as MMPs (Tu and Quan, 2016). In particular, induction of TGF-β signaling by oxidative stress has been associated with increased transcription of collagens and fibronectin (Iglesias-De La Cruz et al., 2001; Zhao et al., 2008; Krstić et al., 2015; Tu and Quan, 2016). Another study, points to the activation of the TNF pathway as important for the transcription of collagen I and III genes, in response to angiotensin II-induced oxidative stress (Sriramula and Francis, 2015), possibly *via* the activation of the NF-κβ transcription factor (Morgan and Liu, 2011). NF-κβ is also activated in response to DNA damage (Janssens and Tschopp, 2006), leading to the transcription of several targets, including ECM related genes (Janssens and Tschopp, 2006; Guo et al., 2021; Figure 3A). NRF2 is another important orchestrator of the oxidative stress response and which acts as a transcription factor (He et al., 2020). This important stress sensor has been shown to be required for the transcriptional regulation of several matrisome genes, including collagen I, III, and laminin α1 chain genes (Hiebert et al., 2018; Hiebert, 2021), raising the possibility that in the presence of oxidative stress, NRF2 may control ECM remodeling (Figure 3A). As mentioned above, the transcriptional regulation of ECM components may also be mediated directly or indirectly *via* the transcription factors p53 and p73, both playing important roles sensing oxidative stress and DNA damage (Holmstrom et al., 2014; Pflaum et al., 2014; Williams and Schumacher, 2016; Figure 3A). Whether these candidate pathways and transcription factors, known to regulate the expression of ECM genes, act in a tissue or disease specific manner, remains to be further explored.

As detailed earlier, several studies point to oxidative stress and DNA damage being a consequence, rather than a cause, of changes in the ECM. In line with this, mutations in genes encoding ECM components or molecules that bridge the ECM to the cell cytoskeleton have been described to lead to mitochondrial dysfunction (Irwin et al., 2003; Millay et al., 2008; Shkryl et al., 2009; Menazza et al., 2010; de Oliveira et al., 2014; Sorato et al., 2014; Fontes-Oliveira et al., 2017; Gremminger et al., 2019; Kölbel et al., 2019), a driving force for ROS accumulation. There are several different mechanisms that can explain why mitochondria may function as an important sensor for ECM changes (De Cavanagh et al., 2009), and therefore, why mitochondrial dysfunction is a hallmark of muscular dystrophies (Cantó-Santos et al., 2020). ECM glycoproteins, including laminins and fibronectin, have been shown to promote mitochondrial function *via* integrin-focal adhesion kinase (FAK) signaling, which culminates in the translocation of phosphorylated STAT3 to the mitochondria (Visavadiya et al., 2016; Figure 3B). Other possible mechanisms are related to the loss of adhesion to the ECM, which changes the cellular uptake of nutrients, namely glucose (Schafer et al., 2009; Eble and De Rezende, 2014). These effects together with the reduction in the activity of phosphatidyl-inositol-3-kinase (PI3K) and Akt, also associated with altered ECM composition, elicits dramatic metabolic changes including decreased activity of the pentose phosphate pathway, which represents an important source of NADPH, a powerful antioxidant molecule (Eble and De Rezende, 2014; Figure 3B). In addition to signaling pathways linking ECM to mitochondria, biomechanical properties of the ECM, such as stiffness (i.e., how rigid the ECM structure is), can impact mitochondrial function (Chen et al., 2021; Figure 3B). High ECM stiffness has been shown to promote mitochondrial fusion and suppress mitochondrial fission downstream of integrin signaling (Chen et al., 2021). The importance of ECM biomechanical properties goes beyond maintaining mitochondrial stability. Low ECM stiffness compromises the DNA damage response, making cells more sensitive to DNA damaging agents (Deng et al., 2020), while high ECM stiffness promotes nuclear rupture also leading to increased DNA damage (Cho et al., 2019; Figure 3B). Altogether, these studies suggest that countering mitochondrial dysfunction, and possibly preventing DNA damage, may be a promising strategy to revert the pathology caused by some mutations in genes encoding ECM components, their receptors or molecules that bridge ECM receptors to the cell cytoskeleton.

In conclusion, future studies are needed to characterize all the players and intermediates involved in the link between oxidative stress/DNA damage and ECM remodeling. Only with this increased knowledge can new candidate pathways and targets be identified. This will be instrumental to establish novel lines of therapy, for diseases where ROS and DNA damage drive ECM remodeling, or where mutations in ECM-related genes elicit an over-accumulation of ROS and DNA damage.

## Author Contributions

SGM and ARC performed the literature review and wrote the manuscript. RZ and ST contributed with active discussion and revision. All authors contributed to the article and approved the submitted version.

## Conflict of Interest

The authors declare that the research was conducted in the absence of any commercial or financial relationships that could be construed as a potential conflict of interest.

## Publisher’s Note

All claims expressed in this article are solely those of the authors and do not necessarily represent those of their affiliated organizations, or those of the publisher, the editors and the reviewers. Any product that may be evaluated in this article, or claim that may be made by its manufacturer, is not guaranteed or endorsed by the publisher.
